# The Impact of Multimodal Prehabilitation on Patient-Reported Outcomes for a Frail Octogenarian Undergoing Multilevel Lumbar Spinal Fusion Surgery: A Case Report

**DOI:** 10.7759/cureus.46836

**Published:** 2023-10-11

**Authors:** Regina Knudsen, Adam Polifka, Keri Ann Markut, Catherine Price, Basma Mohamed

**Affiliations:** 1 Department of Anesthesiology, University of Florida College of Medicine, Gainesville, USA; 2 Department of Neurosurgery, University of Florida College of Medicine, Gainesville, USA; 3 Department of Orthopaedic Surgery and Sports Medicine, University of Florida College of Medicine, Gainesville, USA

**Keywords:** geriatric anesthesia, patient-reported outcomes, frailty, spine fusion, prehabilitation

## Abstract

This case report presents the perioperative optimization pathway of a frail octogenarian who underwent multilevel lumbar spinal fusion surgery. This patient was enrolled in a multimodal prehabilitation program for frail older adults. The multimodal prehabilitation program includes preoperative interventions that prevent further decline in physiological functions before spine surgery. The program focuses on physical exercise, nutritional intervention, and pain neuroscience education. Six weeks postoperatively, clinical and patient-reported outcomes improved in the categories targeted by the preoperative interventions and surgery. This report suggests that prehabilitation is feasible for preoperatively optimizing frail older adults undergoing complex spine surgery.

## Introduction

Due to the exponential increase in the older adult population [1], older adults will increasingly present with symptomatic degenerative spine disease for surgery. Older adults requiring surgery present multiple challenges to the anesthesiology and surgery teams due to multiple comorbidities, cognitive status, functional status, and social capacity [1,2]. Frailty, an age-related decline in physiological reserve, has been associated with poor postoperative outcomes, including increased length of hospital stay, increased risk of non-home discharge, and morbidity [3-5]. Despite the known impact of frailty on postoperative outcomes, very little was found in the literature to evaluate the optimization of frail older adults in preparation for complex spine surgery. This case report presents the impact of multimodal prehabilitation (MPP) in an octogenarian patient on postoperative clinical and patient-reported outcomes after complex spine surgery. The patient was enrolled in an MPP study, which was approved by the University of Florida Institutional Review Board (IRB202000987), and she gave written consent to publish this case report (NCT05034341). A Health Insurance Portability and Accountability Act of 1996 (HIPAA) Authorization form was obtained/completed. This article adheres to the CARE guidelines, and was previously presented as a poster at the 2023 University of Florida College of Medicine Celebration of Research on February 27-28, 2023 and as an abstract and poster at the 2023 University of Florida Department of Anesthesiology Celebration of Research on March 29, 2023.

## Case presentation

An 83-year-old woman with degenerative lumbar scoliosis presented for elective L1-L5 extreme lateral interbody fusion (XLIF) after failing conservative management. Her medical history was significant for diet-controlled diabetes mellitus and hypertension. She denied a history of postoperative cognitive complications or delirium.

The patient was evaluated through our institutional preoperative anesthesia evaluation program for spine surgery patients. The program focuses on medical optimization, nutritional screening, functional assessment including frailty, patient education, and preoperative cognitive screening [6]. She was found to be frail with limited functional capacity. Her Fried Frailty score was 4 out of 5, attributed to exhaustion, slow walking speed, low physical activity, and a weak hand grip. Cognitively, the patient scored normally on general cognitive screeners and average to high average on standardized measures of attention, working memory, verbal learning and memory, and fluency. Neuropsychology highlighted risks for postoperative delirium, which included receiving anticholinergic medication, self-reported high pain levels, and frailty.

Multimodal prehabilitation program (MPP)

The patient elected to partake in an MPP involving exercise, nutrition, and pain neuroscience education (PNE). The physical exercise program, designed by the Department of Physical Therapy in collaboration with the Departments of Anesthesiology and Neurosurgery, included strength training that focused on the functional strengthening of upper and lower extremities and core musculature in frail patients, in addition to aerobic exercises. Physical therapists supervised the physical prehabilitation program, which took place two to three times a week for eight weeks. The nutritional screening included prealbumin and albumin levels. The screening was negative for malnutrition; however, the patient was advised to start targeting a daily protein intake of 1.2 to 1.5 grams/kg body weight per the European Society for Clinical Nutrition and Metabolism Guidelines [7,8]. The chronicity of back pain might result in different coping skills. As a result, PNE included a 20-minute patient education video explaining the nature of spine-related back pain, postoperative expectations, and various strategies to cope with the pain after surgery, as well as encouraging physical activity and emphasizing active patient engagement.

**Table 1:** Patient-Reported Outcomes at Baseline and Postoperatively ^a^ SF-36, Short Form Health Survey Questionnaire; ^b^ ODI, Oswestry Disability Index; ^c^ PROMIS-SF, Patient Reported Outcome Measurement Information System – Short Form. PROMIS scores are given as T-scores.

	Baseline score	6 weeks postoperative score	Interpretation of scores
SF-36^a^ physical functioning	10	15	↑
SF-36 role limitations due to physical health	25	0	↓↓
SF-36 role limitations due to emotional problems	100	100	↑↓
SF-36 energy/fatigue	40	10	↓↓
SF-36 emotional well-being	88	64	↓↓
SF-36 social functioning	62.5	100	↑↑
SF-36 pain	22.5	77.5	↑↑↑
SF-36 general health	75	75	↑↓
SF-36 health change	25	75	↑↑↑
ODI^b^ for low back pain	24	4	↑↑↑↑
PROMIS-29^c^ physical function category	28.7	30.1	↑
PROMIS-29 anxiety category	40.3	56.2	↓
PROMIS-29 depression category	41	55.5	↓
PROMIS-29 fatigue category	48.6	66.6	↓
PROMIS-29 sleep disturbance category	42.2	62.4	↓↓
PROMIS-29 social participation category	39.5	27.5	↓
PROMIS-29 pain interference category	71.6	41.6	↑↑
PROMIS-29 global pain category	8	0	↑↑↑↑

MPP preoperative findings

**Figure 1:**
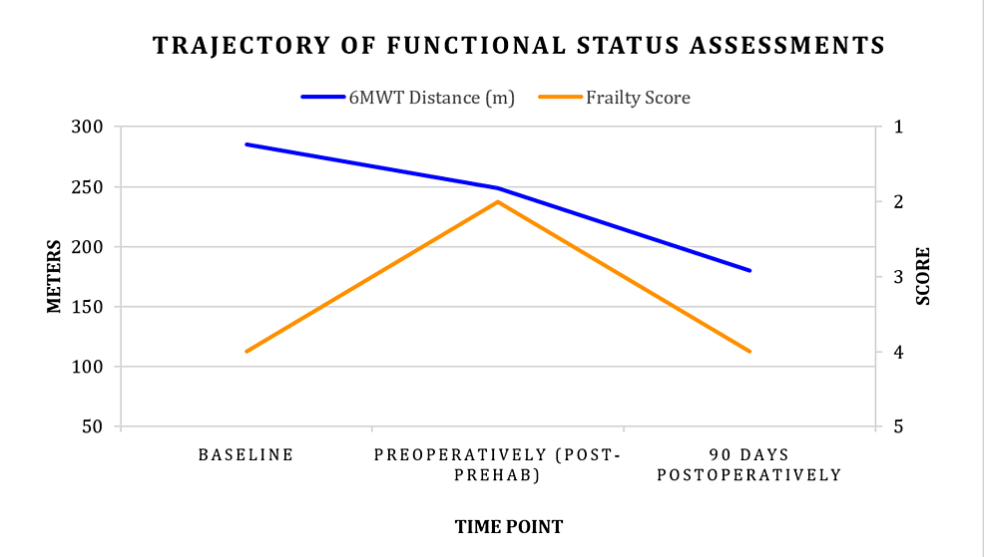
The trajectory of functional status measurements including Fried Frailty and 6-Minute Walk Test (6MWT) Prehabilitation improved this patient’s frailty score in preparation for surgery. However, her frailty score showed no improvement postoperatively. The decline in this patient’s 6MWT was steadily attributed to her chronic pain and postoperative recovery.

Upon starting the prehabilitation program, the patient underwent a consultation with a licensed physical therapist to assess her baseline functional capacity, including muscle strength, range of motion, and flexibility. Her functional gluteal maximus strength was found to be optimal for bed mobility. However, her performance on the Timed Up-and-Go test, Tandem Balance test, and 30-second Sit-to-Stand test showed high fall risk. Self-reported measures of general functioning were reported through the 36-Item Short Form Health Survey (SF-36), Oswestry Disability Index (ODI), and Patient-Reported Outcomes Measurement Information System (PROMIS-29) surveys (see Table 1). A six-minute walk test [9] to assess her functional exercise capacity showed 77% predicted (285 meters) (Figure 1). She underwent physical prehabilitation as recommended. Nutritional protein shakes were provided with instructions. A PNE video was provided for the patient seven days before her surgery.

Perioperative course

On the day of surgery, her perioperative course was uneventful. She received standard anesthesia induction and maintenance. Her perioperative pain management included an intraoperative dose of methadone at 0.2 mg/kg, acetaminophen, and ketorolac. Minimal blood loss was noted during a 380-minute multilevel spine surgery. The patient was transferred to the postanesthesia care unit in stable condition.

Postoperative care included multimodal pain management with average pain scores of 3.6, 0.9, and 2.9 on postoperative days (PODs) 1, 2, and 3, respectively. Her postoperative total morphine milligram equivalent was 7.5 mg total administered POD 1. Otherwise, her postoperative recovery was only complicated by an episode of atrial fibrillation on POD 2, for which she was managed appropriately without impacting her recovery. Of note, the patient was engaged in her care and requested ambulation from the nursing staff 36 hours after surgery. Her hospital stay was seven days, followed by two weeks of inpatient and four weeks of home rehabilitation (twice/week, 60 minutes/session).

MPP six-week outcome

**Figure 2:**
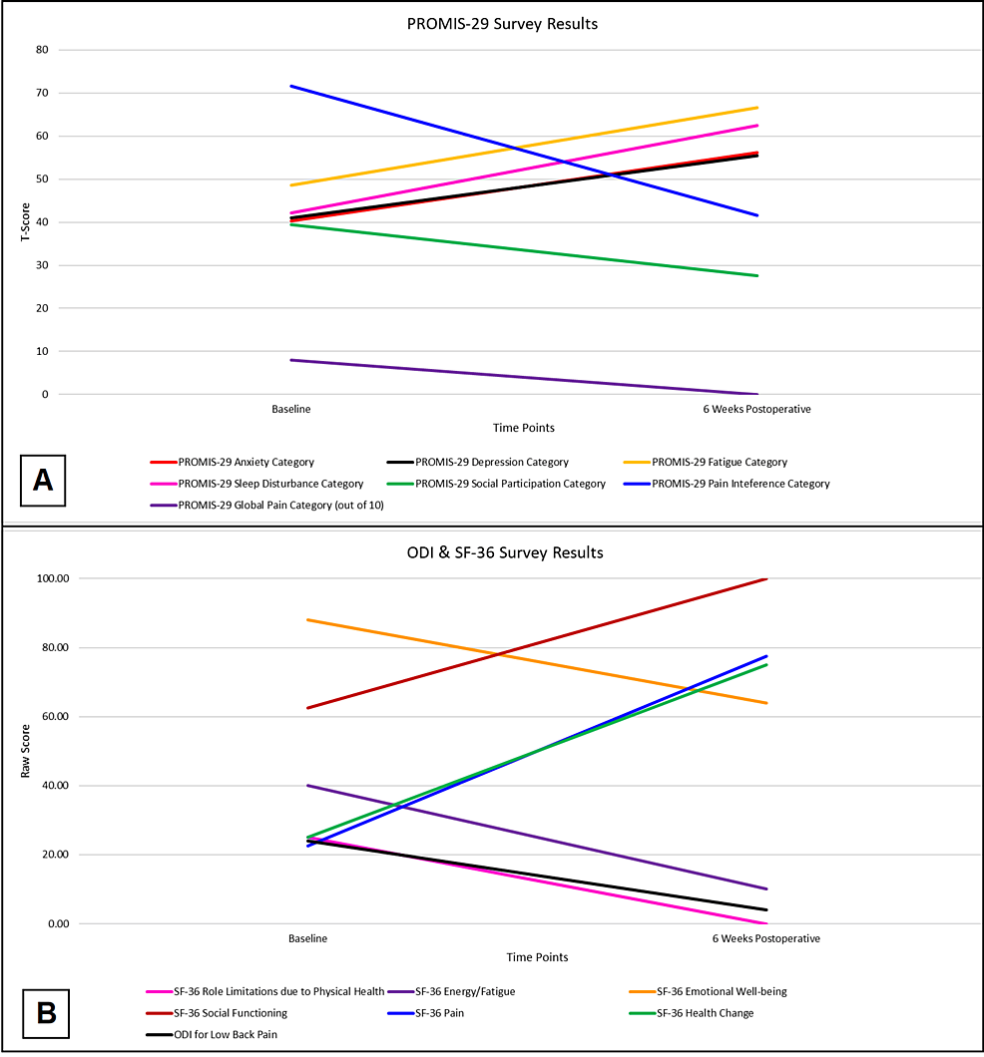
Patient-reported outcomes (A) PROMIS-29^a^ patient-reported outcomes. (B) ODI^b^ and SF-36^c^ patient-reported outcomes. ^a^ PROMIS-29, Patient-Reported Outcomes Measurement Information System. ^b^ ODI, Oswestry Disability Index; ^c^ SF-36, Short Form Health Survey Questionnaire.

Postoperative patient-reported outcome measures, including SF-36, ODI, and PROMIS-29, performed at six weeks showed an improvement in components directly targeted by the prehabilitation program and surgery while showing a decline in the psychological components, including anxiety, depression, and sleep, as shown in Table 1. Her ODI showed improvement in the impact of pain on her social and functional limitations. SF-36 showed improved physical functioning, social functioning, pain, and health change with worsening energy, fatigue, and emotional well-being. PROMIS-29 showed improved physical functioning, pain interference, and global pain. However, it highlighted worsening anxiety, depression, fatigue, sleep disturbance, and social participation (Figure 2).

Patient-reported outcome measures resulted in a neuropsychology referral for cognitive evaluation. Subjectively, the patient did not recall the postoperative ICU stay and “only parts” of rehabilitation. She reported difficulty with sleep and experienced confusion. Objective testing showed a decline on general cognitive screening (pre: 28/30; post: 26/30; Mini-Mental State Examination) with standardized tests half a standard deviation lower for verbal retrieval, processing speed, and working memory. Strengths were retained in her ability to learn new information. The patient reported moderate fatigue (5 out of 10; 10 = worst) but denied pain (0 out of 10) and depression (Patient Health Questionnaire-9 = 1 out of 27 max). Diagnostic considerations included a postoperative neurocognitive disorder. Neuropsychology educated the patient and the family.

## Discussion

This case report documents the feasibility of performing a multimodal prehabilitation program in a frail octogenarian patient and its impact on clinical and patient-reported outcomes. Preoperative to postoperative (six-week) measures showed that the patient experienced reduced pain, improved physical functioning, and improved social engagement. These were domains targeted by the MPP interventions. By contrast, the patient reported increased fatigue and subjective cognitive impairment. Follow-up with Neuropsychology identified mild postoperative cognitive decline and possible postoperative delirium. This case documents (1) the complexity of prehabilitation planning for frail older adults to achieve optimal physical improvements without negatively impacting psychological or cognitive postoperative status and (2) the value of a multidisciplinary approach to the care of the geriatric surgical patient.

Despite the emphasis in the current literature regarding the impact of frailty on postoperative outcomes after spine surgery, there was a paucity in the literature on the role of preoperative optimization of frailty or the impact of prehabilitation on improving postoperative outcomes in spine surgery patients. Yagi et al. evaluated the efficacy of treating frailty, measured as an accumulation of comorbidities, and found no difference in outcomes between treated and untreated frail patients [10].

Prehabilitation interventions, including exercise, nutrition, and psychological components, have been introduced as an optimization intervention for frail cancer patients [8]. When applied before surgery, these interventions aimed to improve the patient's physiological reserve and functional status, which may support enhanced recovery and improve postoperative outcomes. Prehabilitation, in the form of exercise programs, cognitive behavioral therapy (CBT), or PNE, has been studied in spine surgery patients, mainly before surgery for lumbar degenerative spine disease. None of the existing studies focused on frail older adults [11-13]. In a recent systematic review analyzing the impact of current prehabilitation programs on outcomes in patients undergoing spine surgery, the authors could only pool results from studies evaluating CBT. Only one study evaluated the impact of exercise and found a positive effect on short-term outcomes [14]. None of the studies evaluated the impact of prehabilitation on reversing frailty or impacting patient-reported outcomes.

## Conclusions

While significant improvements were seen in multiple patient-reported outcomes in this patient, the main intervention that contributed to these improvements might be surgery, prehabilitation, or both. Frailty has been shown to be associated with adverse outcomes after spine surgery; however, there are no studies or case reports that addressed the role of prehabilitation in optimizing frail older adults electing spine surgery. Future studies should aim to evaluate the impact of prehabilitation on patient-reported outcomes after prehabilitation, before and after surgery. In addition, the choice of nutritional intervention was based on recommendations for cancer patients since there are no recommendations for frail older adults electing spine surgery. Nevertheless, multimodal prehabilitation prepared this patient for a challenging recovery after complex spine surgery. She was actively engaged in her recovery, which may have contributed to improved outcomes.

Despite all the barriers to implementing prehabilitation in this patient population, multimodal prehabilitation can be a feasible modality for preoperatively optimizing frail older adults undergoing complex spine surgery. Instead of declining surgery, spine surgeons might offer prehabilitation as an optimization intervention that may allow frail older adults with spine disease to improve their functional status.
